# The Histopathological Patterns of Ovarian Neoplasms in Different Age Groups: A Retrospective Study in a Tertiary Care Center

**DOI:** 10.7759/cureus.33092

**Published:** 2022-12-29

**Authors:** Nouran H Farag, Zahraa H Alsaggaf, Noha O Bamardouf, Dhuha M Khesfaty, Morouj M Fatani, Maha K Alghamdi, Samah N Saharti

**Affiliations:** 1 Medicine, King Abdulaziz University, Faculty of Medicine, Jeddah, SAU; 2 Dermatology, King Abdulaziz University, Faculty of Medicine, Jeddah, SAU; 3 Internal Medicine, King Abdulaziz University, Faculty of Medicine, Jeddah, SAU; 4 Pathology, King Abdulaziz University, Faculty of Medicine, Jeddah, SAU

**Keywords:** epidemiology, gynecology, histopathological spectrum, ovarian masses, ovarian neoplasms

## Abstract

Objectives

Ovarian cancer is one of the most prevalent neoplasms worldwide and it affects women of all ages. This study aimed to identify the common histopathological patterns of ovarian cancer among different age groups in the western region of Saudi Arabia.

Methods

This was a retrospective study that reviewed all ovarian specimens diagnosed as “ovarian tumors” by the Pathology Department from January 2016 to December 2020 at King Abdulaziz University Hospital, Saudi Arabia. The frequencies of ovarian neoplasm subtypes and their frequencies in different age groups were calculated.

Results

Out of 565 ovarian specimens studied, 63.2% were ovarian neoplasms while 36.8% were non-neoplastic functional cysts. Benign neoplasms 64.4% were more common than borderline 6.2% and malignant ones 29.4% in all age groups, except above the age of 60. Collectively as a category, surface epithelial neoplasms were the most common (59.4%). However, germ cell tumor in the form of mature cystic teratoma was the most common benign neoplasm 33.9% and the most common malignant was serous cystadenocarcinoma (40%).

Conclusion

Documenting new trends of histopathological patterns of ovarian neoplasms helps to detect variation among different age groups and to understand probable predisposing factors. This study found that the percentage of ovarian malignancy has increased over the years in the western region of Saudi Arabia. This signifies the need to increase awareness in order to achieve timely diagnosis and management.

## Introduction

Ovarian neoplasms are one of the most serious health issues that affect women globally, as 313,959 new cases and 207,252 deaths were reported in 2020 [1]. Ovarian cancer is the eighth most common cancer reported in females worldwide, and the eighth cancer-related cause of death in women [1]. In Saudi Arabia, it represents the seventh most common cancer in females [1]. According to a study conducted in 2018, the incidence of ovarian cancer has increased by four-folds in the Saudi population [2].

While ovarian cancer ranks as the third gynecologic cancer following cervical and uterine cancers, it has the least favorable prognosis and the highest death rate [1,3]. The high death rate of ovarian carcinoma is attributed to the silent course of the disease, delayed presentation, and lack of screening programs which lead to its diagnosis during late stages; thereby, naming it the silent killer [4,5].

Ovarian cancer has a wide age of distribution and affects women of all ages but is mostly diagnosed between 55 and 64 [6]. Familial genetic syndromes have shown to be the strongest risk factors influencing ovarian cancer, accounting for 10-12% of all cases [7,8]. Other risk factors include increased age, postmenopausal hormone therapy, and obesity [6,9,10].

A wide spectrum of tumors with different histologies could emerge from the ovaries according to the cell of origin, such as tumors arising from epithelial, sex cord-stromal, germ cells, connective tissue, and metastatic non-ovarian cell [11]. Race, geographic location, and age play a major role in determining which histopathological type is more prominent than the other [12]. A previous study that was published in 2012, conducted in the western region of Saudi Arabia has found the leading benign ovarian neoplasm is serous cystadenoma while the commonest malignant neoplasm is serous cystadenocarcinoma [13]. Similar results were found in studies that were conducted in Pakistan, India, and Sudan [11,14,15].

The aim of this current study is to determine the histopathological patterns of ovarian neoplasms among women in the western region of Saudi Arabia.

## Materials and methods

Ethical consideration

This study was authorized by the institutional review board (IRB) of King Abdulaziz University Hospital (KAUH) (Ref no.). Informed consent was waived due to the retrospective nature of the study.

Study setting and participants

This retrospective descriptive study was conducted from request forms and histopathological reports of all ovarian tumors diagnosed at the Pathology Department, KAUH, a tertiary care center in Jeddah, Saudi Arabia. It was approved by the hospital’s unit of biomedical ethics and research committee. To conduct this study, medical records, constituting the term “ovary,” of 879 females were retrieved from the hospital’s computerized database and were reviewed. The study included all specimens diagnosed as ovarian tumors, both neoplastic, non-neoplastic, and metastatic, collected between January 2016 and December 2020. The diagnosis of the ovarian tumor was based on the histopathological reports issued by the Pathology Department. Both, specimens that were originally diagnosed at the Pathology Department at KAUH and specimens that were referred from different hospitals were included, provided the patient has complete medical records on the KAUH database. Specimens with unremarkable ovarian findings, incomplete or missing patient data were excluded. A total of 565 cases were studied.

Data collection

The data acquired included the patient’s medical record number, name, age, nationality, weight, height, and the patient’s initial presenting complaint. Data from the histopathology reports included specimen source, type of tumor (neoplastic or non-neoplastic), stage, type, and subtype of neoplasm. Based on the WHO classification of ovarian tumors, this study divided them into epithelial (serous, mucinous, endometrioid, clear cell, Brenner, fibroma, adenosarcoma, sero-mucinous carcinoma, and serous cystadenofibroma), germ cell (mature cystic teratoma, immature teratoma, and dysgerminoma), sex cord (fibroma, fibrothecoma, Sertoli cell tumor, juvenile granulosa cell tumor, and adult granulosa cell tumor), and metastasis (uterus, colorectal, appendix, peritoneum, omentum, fallopian tube, breast, bladder, cervix, and lymph node). For descriptive purposes, the age was classified into four categories, less than 20, 20-40, 41-60, and more than 60.

Statistics

The data were gathered and filtered using Microsoft Office Excel 2020 sheet. Statistical analysis was performed using the Statistical Package for Social Sciences (SPSS) version 21 for frequencies. Qualitative data were expressed as numbers and percentages. Quantitative data were reported as measures of central tendencies.

This study aims to demonstrate the histopathological patterns of ovarian neoplasms among women in the western region of Saudi Arabia.

## Results

A total of 565 specimens were reviewed, of which (n=357, 63.2%) were ovarian neoplasms, while (n=208, 36.8%) were non-neoplastic which were excluded from further analysis in this study.

The ages of the study group consisted of less than 20 (n=35, 46.8%), 20-40 (n=234, 76.6%), 41-60 (n=233,23.4%), more than 60 (n=63, 53.2%). Out of these, (n=343, 60.7%) were Saudi, while (n=222, 39.3%) were non-Saudi.

**Figure 1:**
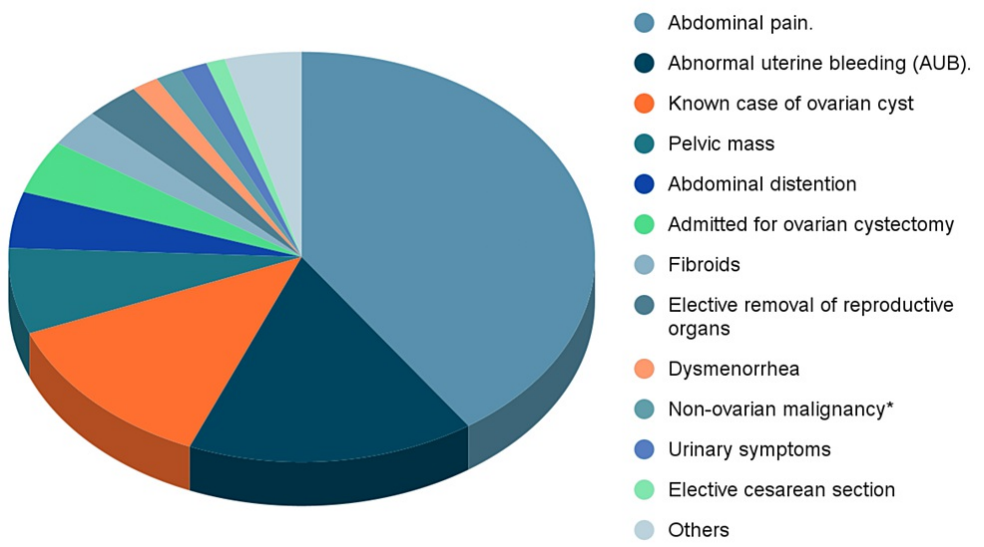
Clinical presentations of cases of ovarian tumors. Non-ovarian malignancy*: known case of non-ovarian malignancy presenting for follow up.

The most common presenting symptom was abdominal pain (n=191, 40.4%) followed by abnormal uterine bleeding (AUB) (n=75, 15.9%) shown in Figure 1.

Across all four age groups, the most common ovarian neoplasm was a surface epithelial tumor (n=212, 59.4%), followed by germ cell tumor (n=82, 23%), sex cord/stromal tumor (n=32, 8.9%), metastatic tumor (n=31, 8.7%), respectively as seen in Table 1.

**Table 1:** Frequency of various types of ovarian neoplasms in different age groups.

Age (years)	Surface epithelial tumors n(%)	Germ cell tumors n(%)	Sex cord/stromal tumors n(%)	Metastatic tumors n(%)	Total n(%)
<20	12 (3.4)	5 (1.4)	4 (1.1)	0 (0.9)	21 (5.9)
20-40	72 (20.2)	56 (15.7)	10 (2.8)	7 (2.0)	145 (40.6)
41-60	96 (26.9)	20 (5.6)	12 (3.4)	12 (3.4)	140 (39.2)
>60	32 (9.0)	1 (0.3)	6 (1.7)	12 (3.4)	51 (14.3)
Total	212 (59.4)	82 (23.0)	32 (9.0)	31(8.7)	357 (100.0)

Out of the 212 surface epithelial tumors, (n=73, 34.4%) were benign serous cystadenoma; (n=8, 3.8%) borderline serous cystadenoma; (n=42,19.8%) serous cystadenocarcinoma; (n=34, 16%) benign mucinous; (n=14, 6.6%) borderline mucinous; (n=3, 1.4%) malignant mucinous; (n=7, 3.3%) benign endometrioid; (n=11, 5.2%) malignant endometrioid; (n=2, 0.9%) clear cell carcinoma; (n=3, 1.4%) Brenner tumor; (n=4, 1.9%) benign mixed epithelial and stromal; (n=2, 0.9%) seromucinous carcinoma; (n=6, 2.8%) serous cystadenofibroma; (n=2, 0.9%) malignant mixed epithelial and stromal.

Germ cell tumors constituted 82 specimens of which n=78, 95.1% were mature cystic teratoma; (n=2, 2.4%) immature teratoma; (n=2, 2.4%) dysgerminoma.

Of all ovarian neoplastic lesions, tumors originating from the sex cord were 32. Out of the 32, (n=17, 53.1%) fibroma; (n=6, 18.8%) fibrothecoma; (n=2, 6.3%) Sertoli-Leydig cell tumor; (n=2, 6.3%) juvenile granulosa cell tumor, (n=5, 15.6%) adult granulosa cell tumor.

Across all age groups, except those above 60 years, benign ovarian tumors were the most common (n=230, 64.4%), followed by malignant tumors (n=105, 29.4%), and borderline tumors (n=22, 6.2%).

In the age group above 60 years, malignant tumors were the most common (n=30, 58.8%), followed by benign (n=19, 37.3%), and borderline (n=2, 3.9%) (Table 2).

**Table 2:** Frequency of benign, borderline, and malignant ovarian tumors in different age groups.

Age (years)	Benign n (%)	Borderline n (%)	Malignant n (%)	Total n (%)
<20	15 (4.2)	2 (0.6)	4 (1.1)	21 (5.9)
20-40	115 (32.2)	11 (3.1)	19 (5.3)	145 (40.6)
41-60	81 (22.7)	7 (2.0)	52 (14.6)	140 (39.2)
>60	19 (5.3)	2 (0.6)	30 (8.4)	51 (14.3)
Total	230 (64.4)	22 (6.2)	105 (29.4)	357 (100)

Out of 230 benign ovarian neoplasms, the most common benign was germ cell tumor in the form of mature cystic teratoma (n=78, 33.9%), followed by surface epithelial tumors in the form of serous cystadenoma (n=73, 31.7%), and mucinous cystadenoma (n=34, 14.8%), fibroma (n=17, 7.4%), endometrioid (n=7, 3%), serous cystadenofibroma (n=6, 2.6%), fibrothecoma (n=6, 2.6%), mixed epithelial and stromal (n=4, 1.7%), Brenner tumor (n=3, 1.3%), and Sertoli-Leydig cell tumor (n=2, 0.9%), respectively (Table 3).

**Table 3:** Frequency of benign ovarian neoplasm in different age groups.

Age (years)	<20 n (%)	20-40 n (%)	41-60 n(%)	>60 n(%)	Total n(%)
Serous cystadenoma	5 (2.2)	27 (11.7)	36 (15.7)	5 (2.2)	73 (31.7)
Mucinous cystadenoma	4 (1.7)	16 (7.0)	11 (4.8)	3 (1.3)	34 (14.8)
Endometrioid	0 (0.0)	5 (2.2)	1 (0.4)	1 (0.4)	7 (3.0)
Mixed (Epithelial and stromal)	0 (0.0)	2 (0.9)	1 (0.4)	1 (0.4)	4 (1.7)
Serous cyst adenofibroma	1 (0.4)	3 (1.3)	1 (0.4)	1 (0.4)	6 (2.6)
Brenner tumor	0 (0.0)	1 (0.4)	1 (0.4)	1 (0.4)	3 (1.3)
Mature cystic teratoma	4 (1.7)	53 (23.0)	20 (8.7)	1 (0.4)	78 (33.9)
Fibroma	1 (0.4)	5 (2.2)	5 (2.2)	6 (2.6)	17 (7.4)
Fibrothecoma	0 (0.0)	2 (0.9)	4 (1.7)	0 (0.0)	6 (2.6)
Sertoli-Leydig cell tumor	0 (0.0)	1 (0.4)	1 (0.4)	0 (0.0)	2 (0.9)
Total	15 (6.5)	115 (50.0)	81 (35.2)	19 (8.3)	230 (100.0)

Out of the 357 ovarian neoplasms, 22 borderline tumors were identified; 14 of which were mucinous, and eight were serous surface epithelial.

Out of the 105 malignant ovarian tumors, the most common was serous cystadenocarcinoma (n=42, 40%), followed by metastatic carcinoma (n=31, 29.5%), endometrioid malignant tumor (n=11, 10.5%), adult granulosa cell tumor (n=5, 4.8%), mucinous cystadenocarcinoma (n=3, 2.9%), clear cell carcinoma (n=2, 1.9%), serous-mucinous carcinoma (n=2, 1.9%), mixed epithelial and stromal (n=2, 1.9%), immature cystic teratoma (n=2, 1.9%), dysgerminoma (n=2, 1.9%), juvenile granulosa cell tumor (n=2, 1.9%), and malignant Brenner tumor (n=1, 1%), respectively (Table 4).

**Table 4:** Frequency of malignant ovarian neoplasms in different age groups.

Age (years)	<20 n(%)	20-40 n(%)	41-60 n(%)	>60 n(%)	Total n(%)
Serous cystadenocarcinoma	0 (0.0)	5 (4.8)	23 (21.9)	14 (13.3)	42 (40.0)
Mucinous cystadenocarcinoma	0 (0.0)	0 (0.0)	3 (2.9)	0 (0.0)	3 (2.9)
Endometrioid malignant tumor	0 (0.0)	0 (0.0)	7 (6.7)	4 (3.8)	11 (10.5)
Clear cell carcinoma	0 (0.0)	1 (1.0)	1 (1.0)	0 (0.0)	2 (1.9)
Serous-mucinous carcinoma	0 (0.0)	1 (1.0)	1 (1.0)	0 (0.0)	2 (1.9)
Malignant Brenner tumor	0 (0.0)	0 (0.0)	1 (1.0)	0 (0.0)	1 (1.0)
Mixed epithelial and stromal	0 (0.0)	0 (0.0)	2 (1.9)	0 (0.0)	2 (1.9)
Immature cystic teratoma	1 (1.0)	1 (1.0)	0 (0.0)	0 (0.0)	2 (1.9)
Dysgerminoma	0 (0.0)	2 (1.9)	0 (0.0)	0 (0.0)	2 (1.9)
Juvenile granulosa cell tumor	2 (1.9)	0 (0.0)	0 (0.0)	0 (0.0)	2 (1.9)
Adult granulosa cell tumor	1 (1.0)	2 (1.9)	2 (1.9)	0 (0.0)	5 (4.8)
Metastatic carcinoma	0 (0.0)	7 (6.7)	12 (11.4)	12 (11.4)	31 (29.5)
Total	4 (3.8)	19 (18.1)	52 (49.5)	30 (28.6)	105 (100.0)

**Figure 2:**
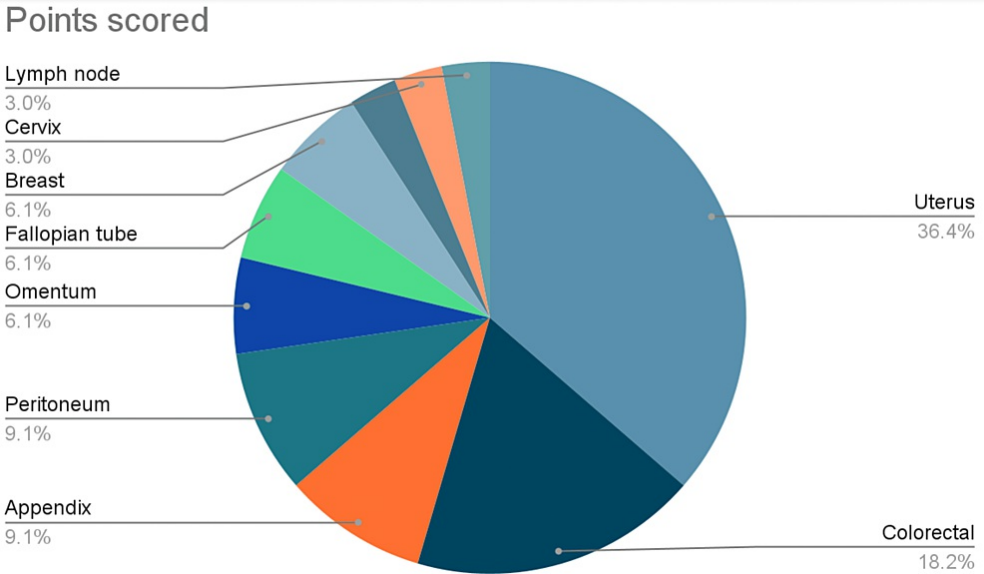
Site of metastasis.

The most common sites of metastasis are the uterus (n=12, 36.4%) followed by the colorectal (n=6, 18.2%), and appendix (n=3, 9.1%) as shown in Figure 2.

## Discussion

A total of 565 ovarian specimens were reviewed; out of which, 357 (63.2%) were neoplastic, while 208 (36.8%) were non-neoplastic. These results were compared to those of another study conducted at the same tertiary care hospital in Saudi Arabia, in which Abdullah et al reported that 382 (61.8%) of 618 ovarian lesions were neoplastic [13]. It should be noted that even though there is a 10-year gap between the previously mentioned study and this current one, neoplastic lesions are still outweighing non-neoplastic lesions in number.

Moreover, an earlier study that was carried out at KAUH and dates back 20 years from the current one revealed non-neoplastic lesions to be of majority (47.5%) and neoplastic lesions to only comprise 29.7% of the population sample [16]. This shows a trend of decreasing number of non-neoplastic ovarian cysts and increasing number of neoplasms over the years. This may be attributed to the fact that simple cysts are diagnosed radiologically and managed conservatively with unusual need to be biopsied, unless there were findings suspicious for malignancy or clear indications for surgical intervention [17]. Therefore, this can make simple cysts less likely to appear on pathology reports. We also assume that this may demonstrate the rising level of awareness about disease and the readily available access to healthcare services, which altogether can encourage patients to seek medical help more than they have had in prior decades and thus increase the chance for established diagnosis [18].

Out of all neoplastic lesions, our results show that benign ovarian neoplasms were 64.4%, while the percentages of borderline and malignant neoplasms were 6.2% and 29.4%, respectively. Yousif et al. had approximately similar findings in their patient population, which were 63% for benign, 7% for borderline, and 30% for malignant [19]. Abdullah and Bondagji found that the percentages of benign, borderline, and malignant neoplasms were 72.8%, 5.2%, 22%, respectively [13]. While the percentages found in the aforementioned study is close to what our study has found, it can be seen that the percentage of malignant neoplasm has increased over the years in the western region.

This study found that the most common benign neoplasm was mature cystic teratoma (33.9%), followed closely by serous cystadenoma (31.7%). There is a consensus that mature cystic teratoma is the most common ovarian neoplasm [20]. However, some studies, such as the one done by Abdullah and Bondagji in Saudi and Sawant and Mahajan in India, illustrate that the most prevalent benign tumor was serous cystadenoma, with a percentage of (44.6%) and (54.5%), respectively [11,13].

As for the most common malignant tumor, it was found to be serous cystadenocarcinoma (40%). This malignant tumor was consistently found as the most frequent primary ovarian malignancy across multiple studies done in Saudi, India, and Sudan [11,13,19,21]. Additionally, a study has found that serous carcinomas were the most prevalent internationally; in Europe, the Americas, Australia, and some Asian countries [3].

Some studies, such as the one done by George and Shaw have found an association between serous ovarian cancer and BRCA gene mutation [22]. According to a recent study done by Agha et al., BRCA mutation was found in 41% of Saudi women diagnosed with ovarian cancer [23]. While assessing the frequency of BRCA mutation was not within the scope of this study, we hypothesize that the increase in the prevalence of ovarian malignancy in Saudi for the past 10 years could possibly be attributed to the high prevalence of BRCA mutation.

We found that benign ovarian neoplasms were the most common among all age groups except for the age group above 60, which had a predominance of malignant ovarian neoplasms. In a similar study, Kheiri et al. reported that most patients (66%) presenting with malignant ovarian neoplasms were older than 50; meanwhile, malignancy was less frequent in patients younger than 40 (14%) [21]. In addition, The American Cancer Society further reinforced the fact that malignant ovarian neoplasms are scant among women younger than 40 years of age, and that 50% of ovarian malignancies are discovered in women over 63 years old [24]. Therefore, we conclude that benign tumors are generally more common than malignant tumors; however, there is a higher chance of malignancy with increasing age, which can be attributed to the effect of aging on the replicative quality of cells. This is known as cellular senescence, a permanent halt of the cell cycle due to aging or buildup of damaged DNA in those cells. The process is also influenced by senescence-associated secretory phenotype (SASP), a phenotype that promotes senescent cells to secrete pro-inflammatory mediators that aim to suppress tumors and stop the progression of abnormal cells [25]. Paradoxically, SASP can contribute to the development of age-related diseases such as cancer due to the immunosuppressive environment that it creates [26].

A common trend for histopathological typing was detected across all age groups, with the most common type being surface epithelial tumors (59.4%), followed by germ cell tumors (23%). These results were also found in other previous studies done in Saudi Arabia, India, Sudan, and Nepal. [11,13,16,21,27]. It is worth noting that surface epithelial malignant neoplasms were not identified in the age group below 20. This is due to both the rare nature of serous carcinomas in the first two decades of life and the fact that mucinous carcinomas usually present in the fifth decade [27]. Only four malignant cases were identified under 20 years of age, of which two were juvenile granulosa cell tumors, one adult granulosa cell, and one immature cystic teratoma. This finding aligns with those of previous studies, and it further supports the hypothesis that malignancy is related to older age [27,28]. A study that was conducted in Nepal showed that the entirety of ovarian malignancies detected below 20 years old were of germ-cell origin [27]. For the benign entity, we established that the most common one in this age group was serous cystadenoma.

In the age group 41-60, the most common benign neoplasm was serous cystadenoma, and the most common malignant neoplasm was serous cystadenocarcinoma. This was similar to what Abdullah and Bondagji had found (taking into consideration the difference in age group classification between their study and the current one). They also found that the same results were shared between the two age groups, 21-50 and >51 years old. However, this study found that the age group above 60 had the most common benign and malignant neoplasms as fibroma and serous cystadenocarcinoma, respectively.

Mature cystic teratoma was the most prevalent benign neoplasm in the age group 20-40, which concurs with what Jha and Karki had found [27]. Furthermore, the most common malignant ovarian neoplasm in this age group was rather metastatic than primary in origin.

Out of all ovarian neoplasms, metastatic ovarian tumors constituted 8.7%. In the United States, metastasis represents 8% of the masses in the adnexa in women undergoing surgery, which is similar to our percentage [20]. A study done in Sudan, found the percentage of metastatic tumors in their population to be 10.4% [21]. Abdullah and Bondagji reported a lesser percentage of metastatic tumors (3.4%), which raises the question of why the percentage of metastatic tumors seemed to have increased over the years in Saudi [13].

In our study, metastasis was mostly uterine in origin (36.4%), followed by colorectal and appendiceal cancers (27.3%). Unlike our study, the Sudanese study states that the origin of metastasis was mostly colonic or appendicular in origin [21]. Interestingly, neither study has listed uterine cancer as a source of metastasis. According to Nucci, the most frequent source of metastasis to the ovaries are colorectal cancers, and that in general, cancers that do not originate from the female genital tract are 11 times more common than those from gynecological organs [20].

Colorectal cancer has a higher incidence in Saudi women than uterine cancer according to the Saudi cancer registry [29]. However, there is no national screening program for colorectal cancer in Saudi [30]. Therefore, the incidence of colorectal cancer, and by extension, its metastases to the ovaries could be underestimated, which may provide an explanation for the contrast found between studies.

To our knowledge, this study has met its goals and had a large sample size, yet some limitations should be highlighted. It is a retrospective study conducted at a single center in the western region. It assessed the histopathological distribution of ovarian neoplasms among the western region population as a whole, rather than focusing on the patients’ racial backgrounds, which could act as a cofounder for our results. Due to its retrospective nature, it has all the limitations inherent to retrospective studies including incomplete data in some patients and selective bias. In addition, the BRCA mutation status of patients could not be detected. Further studies are encouraged to know the cause for increased prevalence of ovarian malignancy.

## Conclusions

In conclusion, benign ovarian neoplasms were the most common in all age groups except in women above the age of 60. This concludes that with increasing age, the percentages of malignant ovarian neoplasms tend to increase. In addition, there’s a noticeable increase in the rate of ovarian cancer within the western region of Saudi Arabia. Therefore, studying the factors that could possibly have contributed to this increase in prevalence and their association with the histopathological types is crucial.
